# Prevalence and Associated Factors for Elevated Depressive Symptoms in 386,924 Primary Students during the COVID-19 Pandemic Normalization in China

**DOI:** 10.3390/ijerph19063406

**Published:** 2022-03-14

**Authors:** Yuan Xue, Qingqing Xu, Juan Wang, Hualiang Lin, Chongjian Wang, Xiaomin Lou, Cuiping Wu, Zhenxing Mao, Xiaoli Fu

**Affiliations:** 1 Department of Epidemiology and Biostatistics, College of Public Health, Zhengzhou University, Zhengzhou 450001, China; xueyuansnow@163.com (Y.X.); xuqingqing9791@163.com (Q.X.); wangjuan2333@163.com (J.W.); tjwcj2005@126.com (C.W.); louxm@zzu.edu.cn (X.L.); wucuiping@zzu.edu.cn (C.W.); maozhr@gmail.com (Z.M.); 2 Department of Epidemiology, School of Public Health, Sun Yat Sen University, Guangzhou 510080, China; linhualiang@mail.sysu.edu.cn

**Keywords:** depressive symptoms, COVID-19, China, students, factors, prevalence

## Abstract

We aimed to assess the prevalence of elevated depressive symptoms and its associated factors during the coronavirus disease 2019 (COVID-19) pandemic among primary students in China. We included 386,924 students aged 6–12 years from three cities in Henan province, China, over the period 21–27 May 2021. The overall prevalence of depressive symptoms was 5.8%. Participants with high depressive symptoms were more likely to be senior urban primary students, and exhibited an insignificant increase in hand washing frequency, non-mask wearing behavior, higher error rates of cognition tests, and greater levels of worry and fear. The associated factors for high depressive symptoms were found to include age, sex, grade, location, worry level, fear level, cognitive status, and change in lifestyle after gaining knowledge about COVID-19. Our results suggest that governments need to focus on factors affecting the mental health of school-age children while combating COVID-19, as it would facilitate better decision making on the international and national level.

## 1. Introduction

Since its outbreak, coronavirus disease 2019 (COVID-19) has quickly become a global health threat and caused unprecedented economic devastation [1]. To impede the spread of the virus, the Chinese government implemented unprecedented strict quarantine measures, prolonged social isolation orders, and closed schools and universities [2]. Continuous stressors easily affect children and adolescents during the sensitive developmental period; thus, more attention should be paid to their mental health during and after the pandemic [3,4]. The epidemic prevention and control work has not stopped, but has shifted to normalization. Normalization means strictly controlling epidemic prevention and control as a daily strategic task, and preparing for a long battle while promoting the full restoration in production and life order. In essence, it means to strike a balance between the “relaxation” of work resumption and the “tightness” of epidemic prevention and control, and adhere to the COVID-19 prevention and control battle for a long time. In this study, we have focused on the prevalence and associated factors for elevated depression symptoms among primary students during the COVID-19 pandemic normalization.

Depression is one of the common mental disorders affecting children and adolescents [5]. Although clinical depression is prevalent in only 1.3% of children and adolescents in China [6], depressive symptoms are relatively common in school-aged children [7]. Because the prevalence of depression usually increases substantially over adolescence, depression in primary students has often been overlooked [8]. Twenty Chinese studies reported a pooled depression prevalence of 45% in patients with COVID-19 from China, while the depression prevalence for Italy, Ecuador, and Iran was 38%, 60%, and 38%, respectively, as reported by one study in each subgroup [9]. Children and adolescents may be more susceptible than adults to mental disorders during the COVID-19 pandemic [10]. A recent study reported that the prevalence of anxiety symptoms among junior and senior high school students in China was 9.89% during the COVID-19 epidemic. Indeed, the psychological impact of the outbreak and resultant psychological crisis was greater in children, and resulted in numerous psychological problems, such as anxiety and depression [11]. A previous study conducted in China has suggested that, at the beginning of the outbreak, an increase in the prevalence of mental disorders in students was associated with COVID-19 [12], though the full impact is presently unknown [13].

Therefore, in order to assist government agencies and health care professionals to safeguard the psychological well-being of school children during the spread of COVID-19 in China and different parts of the world, we assessed the prevalence of elevated depressive symptoms in primary students and identified the potential associated factors for depressive symptoms.

## 2. Materials and Methods

### 2.1. Study Participants

We performed a cross-sectional study to investigate the associated factors for the COVID-19 pandemic normalization on high depressive symptoms in children from 21 May 2021 to 27 May 2021. We recruited 6–12-year-old elementary students using a cluster sampling method from the Zhengzhou, Xinxiang, and Xinyang cities in Henan province, China, and invited them to participate in an online survey through an online survey platform (“SurveyStar”, Changsha Ranxing Science and Technology, Shanghai, China); 438,978 participants were recruited. These regions are representative of the overall conditions in Henan province. We excluded the data of participants whose age was <6 years or >12 years or those who took ≤100 s to fully respond to the questions (*n* = 52,054). After this exclusion process, a total of 386,924 students aged 6–12 years were included in the final analysis.

### 2.2. Data Collection

A standard questionnaire was used to collect data regarding the demographics (age, sex, grade, and residential location) and cognitive status, and determine pandemic characteristics of COVID-19. It included questions such as “will it be passed from person to person”, “route of transmission”, and “quarantine for several days after exposure”, and assessed the mental state (worry and fear), change in lifestyle after gaining knowledge regarding the pandemic (number of hand washes and mask wearing status), and high depressive symptoms. The grades were classified into three levels, i.e., 1–2, 3–4, and 5–6. Residential locations were categorized as cities, rural-level cities, and country-level cities. The cognitive status was classified into three levels (all correct, not all correct, and all wrong) [13]. The worry and fear levels were categorized as high, moderate, and low, based on the score calculated using the 5-point Likert scale [14]. A significant increase in the number of hand washes was identified by whether participants answered yes or no to the relevant question. Mask wearing status was determined by whether participants answered “always”, “when in crowed places or on public transportation”, “occasionally”, and “never”.

Elevated depressive symptoms were assessed using the [15] Children’s Depression Inventory (CDI) [16], a widely used self-report measure for 6–18-year-old children and adolescents. There were 27 items in total, and each item included 3 statements. The participant was asked to select the appropriate option applicable for the past two weeks; the options were occasionally (0), often (1), and always (2). The higher the scores, the more severe the elevated depressive symptoms. The score range was 0–54, and the cut-off value was 19 [17].

### 2.3. Statistical Analysis

Continuous variables were expressed in terms of mean values (standard deviation) and compared using *t*-tests, while categorical variables were expressed as a percentage and compared using the chi-squared test. Logistic regression analysis was used to estimate the association between mixed factors and the risk of elevated depressive symptoms. Odds ratio (OR) and 95% confidence interval (CI) values for high depressive symptoms were reported in separate models. Multivariable adjustment modeling was performed as follows: model 1 was adjusted for age, sex, grade, and location; model 2 was adjusted for the parameters listed for model 1 and the level of worry and fear, cognitive status, and GAD-2 scores. Statistical analyses were performed using SPSS software, version 21.0 (SPSS Inc., Chicago, IL, USS) and SAS V.9.1 (SAS Inst., Cary, NC, USA). *p* values of <0.05 in the two-sided test were considered to be statistically significant.

## 3. Results

### 3.1. Basic Characteristics

Out of 386,924 participants, 205,967 girls and 180,957 boys who were 6–12 years old were invited to participate in the online survey during the COVID-19 outbreak in China. The study population included 22,602 patients with elevated depressive symptoms (5.84%). Participants with elevated depressive symptoms were more likely to be female, older, urban students, non-mask wearers, and exhibit a non-significant increase in the number of hand washes, higher error rate of cognition test, and higher level of worry and fear (Table 1).

### 3.2. Prevalence of High Depressive Symptoms

**Figure 1:**
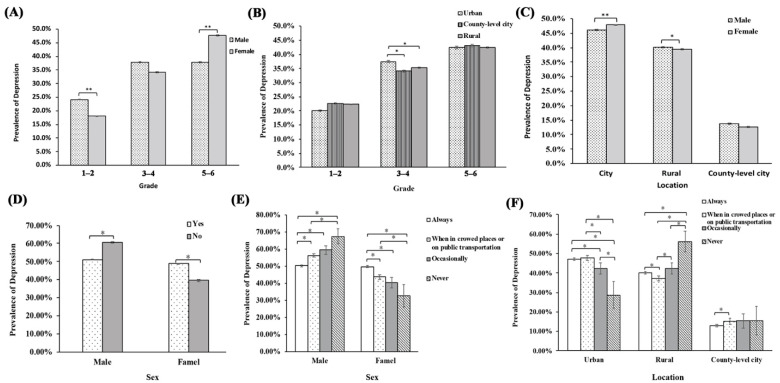
Prevalence and standard error of elevated depressive symptoms in participants by grade and sex (**A**), grade and location (**B**), location and gender (**C**), sex and hand washing status (**D**), sex and mask wearing status (**E**), location and mask wearing status (**F**); *: *p* <0.05; **: *p* < 0.001.

Through further analysis, we found that the prevalence was increased with an increase in the grade in both sexes (*p* < 0.001). Figure 1A shows that the prevalence of grades 1–2 in males was higher than that in females (24.2% vs. 18.1%). In contrast, the prevalence of grades 5–6 in females was higher than that in males (37.9% vs. 47.6%). The prevalence of grades 3–4 was higher in urban areas than in rural-level and county-level cities (37.4% vs. 35.2% vs. 34.1%), while that for other grades was lower (Figure 1B). As shown in Figure 1C, the prevalence of high depressive symptoms was higher in girls (47.0%) than in boys (46.1%) in the urban, while the prevalence was higher in boys in rural-level and country-level cities, i.e., 40.2% vs. 39.4% and 13.7% vs. 12.7% (boys vs. girls), respectively. The prevalence was higher in boys when the number of hand washes did not increase, while a contrasting scenario was observed in girls (Figure 1D). The prevalence of non-masking behavior was higher for girls in rural-level cities, while that for boys in rural areas was low (Figure 1E,F).

### 3.3. Associated Factors for Elevated Depressive Symptoms

Table 2 presents the association between the characteristics of study participants and elevated depressive symptoms using multivariable logistic regression analysis. The ORs (95% CIs) of the oldest group were 3.05 (2.94, 3.17) and 2.96 (2.85, 3.07), compared to those of the youngest group in the crude and full-adjusted model. Compared to males, the odds of elevated depressive symptoms in females were increased by 10% (OR: 1.10, 95% CI: 1.07–1.13). Meanwhile, after full adjustment, the OR (95% CI) of students from rural areas was 1.35 (1.31, 1.39) compared to that of students from cities. Students with the worst cognitive status during the COVID-19 pandemic had a significantly increased risk of elevated depressive symptoms, compared to that of participants with the best cognitive status (OR: 4.03, 95% CI: 3.50, 4.65). In addition, an insignificant increase in the number of hand washes and non-mask wearing behavior could increase the risk of elevated depressive symptoms (OR: 4.95, 95% CI: 4.62, 5.32; OR: 8.16, 95% CI: 6.29, 10.59).

## 4. Discussion

Our findings demonstrate that the prevalence of elevated depressive symptoms in students is different (5.8%) from that reported in a previous study (1.3%) [6]. This may be attributable to their sudden inability to participate in numerous activities that provide structure, meaning, and daily rhythm, such as school, extracurricular activities, social interactions, and physical activities, owing to the outbreak. Depressive symptoms in school children are associated with certain socio-demographic factors, such as sex [18], age [19], anxiety [20], and academic stress [18].

In this study, a higher rate of prevalence in the lower grades with elevated depressive symptoms was observed in boys than in girls; in contrast, elevated depressive symptoms are more likely to occur in females in the higher grades. These findings regarding sex-related differences in depressive individuals are aligned with those in prior studies [21,22,23]. These sex-related differences could be attributable to brain development and sex hormones [24,25,26]. Skills, self-control, and social maturity typically improve over the course of development, which may result in decreased diagnoses in boys, while hormonal and social changes during adolescence may trigger depression in girls [27].

Our study showed that the highest prevalence of elevated depressive symptoms was observed in participants who lived in cities. Moreover, higher rates of mental disorders were observed in children and adolescents from developed areas, compared to students living in less developed areas [21]. Children living in cities were subjected to increased exposure to media, which could increase anxiety and stress responses, and further result in negative effects on mental health [28,29]. Meanwhile, the prevalence of elevated depressive symptoms was also significantly different in different sexes in the urban and rural areas. The findings that girls and children in urban environments are showing more symptoms of depressions is in line with a substantial amount of earlier research. This suggests that the psychological health of boys in rural areas and girls in urban areas requires more attention.

Moreover, the present study highlighted that the extent of elevated depressive symptoms increased with a neglect of behavioral changes. Elevated depressive symptoms can be alleviated upon taking the correct response measures during the pandemic. Notably, the association between elevated depressive symptoms and increased hand washing frequency and wearing of masks was different in boys and girls. This could be attributed to the differences in public awareness and education, especially because girls were more sensitive. Similarly, differences were observed in the population in urban and rural areas. Therefore, differences in sex and region should be considered during future attempts to enhance public awareness and education. By focusing upon public awareness and education, children can achieve a more comprehensive understanding regarding COVID-19, and protect themselves by developing hygienic habits such as hand washing and mask wearing. This would reduce the influence of COVID-19 on elevated depressive symptoms among children.

Our study is a comparatively large sample study on the prevalence of elevated depressive symptoms in primary students. Second, Henan province, which has the largest educated population in China, has a border with Hubei province. The close contact between the two provinces reflects the level of elevated depressive symptoms in students from these provinces during the COVID-19 pandemic. Third, we used a standardized questionnaire (CDI) to diagnose elevated depressive symptoms. Finally, we excluded the participants who did not meet the requirements of this study, to ensure that our assessments were realistic.

Nevertheless, several limitations are associated with this study. First, although we adjusted many covariates, we still could not rule out the possibility of other potential confounding factors. Second, prevalence may be skewed upon assessing the symptoms of depression using self-reported scales instead of clinical interviews. However, we used the CDI scale, which is a simple and highly effective self-assessment tool for evaluating elevated depressive symptoms. Third, the cognitive status reflects the participants’ understanding of the pandemic characteristics of COVID-19, but the effectiveness of the cognitive status is not guaranteed. Fourthly, the cross-sectional study was unable to establish a causal relationship between exposure and outcome. Finally, as the subjects in this study were primary students, it might not be possible to extend our findings to students from other grades.

## 5. Conclusions

In summary, the prevalence of elevated depressive symptoms among Chinese primary students, especially grades 5–6 and 3–4 students or students living in rural areas, was not optimal during the COVID-19 pandemic normalization. Factors such as age, sex, grade, location, cognitive status, and change in lifestyle (increase in the number of hand washes and mask wearing status) after gaining knowledge regarding the pandemic should be considered during the overall management of elevated depressive symptoms.

## Figures and Tables

**Table 1:** Characteristics of the study participants by elevated depressive symptoms status.

Characteristics	All Participants *n* = 386,924	Non-Depressive Symptoms *n* = 364,322	Elevated Depressive Symptoms *n* = 22,602	***p*-** **Value**
Age (years)	9.59 ± 1.61	9.27 ± 1.77	9.42 ± 1.79	<0.001
Sex, *n* (%)				<0.001
Male	205,967 (53.2)	194,298 (53.3)	11,669 (51.6)	
Female	180,957 (46.8)	170,024 (46.7)	10,933 (48.4)	
Grade, *n* (%)				<0.001
1–2	158,516 (41.0)	153,704 (42.2)	4812 (21.3)	
3–4	134,302 (34.7)	126,137 (34.6)	8165 (36.1)	
5–6	94,106 (24.3)	84,481 (23.2)	9625 (42.6)	
Location, *n* (%)				<0.001
Urban	20,3506 (52.6)	192,896 (52.9)	10,610 (46.9)	
Rural	123,909 (32.0)	114,904 (31.5)	9005 (39.8)	
Country-level city	59,509 (15.4)	56,522 (15.5)	2987 (13.2)	
Worried level, *n* (%)				<0.001
High	246,932 (63.8)	230,130 (63.2)	16,802 (74.3)	
Moderate	83,110 (21.5)	79,227 (21.7)	3883 (17.2)	
Low/none	56,882 (14.7)	549,65 (15.1)	1917 (8.5)	
Fear level, *n* (%)				<0.001
High	166,584 (43.1)	153,207 (42.1)	13,377 (59.2)	
Moderate	138,280 (35.7)	132,129 (36.3)	6151 (27.2)	
Low/none	82,060 (21.2)	78,986 (21.7)	3074 (13.6)	
Cognitive status, *n* (%)				<0.001
All correct	98,820 (25.5)	94,276 (25.9)	4544 (20.1)	
Not all correct	286,775 (74.1)	269,051 (73.8)	17,724 (78.4)	
All wrong	1329 (0.3)	995 (0.3)	334 (1.5)	
Significant increase in hand washing frequency, *n* (%)				<0.001
Yes	382,271 (98.8)	361,042 (99.1)	21,229 (93.9)	
No	4653 (1.2)	3281 (0.9)	1373 (6.1)	
Mask wearing status, *n* (%)				<0.001
Always	324,871 (84)	306,678 (84.2)	18,193 (80.5)	
When in crowed places or on public transportation	59,328 (15.3)	55,714 (15.3)	3614 (16.0)	
Occasionally	2424 (0.6)	1786 (0.5)	638 (2.8)	
Never	301 (0.1)	144 (0.0)	157 (0.7)	

Continuous variables are presented in mean (standard deviation) and compared by *t*-test; categorical variables are expressed in number (percentage) and compared by chi-squared test.

**Table 2:** Independent association between characteristics of study participants and high depressive symptoms during the COVID-19 pandemic in China.

Characteristics	Crude		Model 1		Model 2	
	OR (95% CI)	*p*-Value	OR (95% CI)	*p*-Value	OR (95% CI)	*p*-Value
Age (years)						
6–8	1.00 (ref)		1.00 (ref)		1.00 (ref)	
8–10	1.48 (1.41, 1.55)	<0.001	1.48 (1.41, 1.56)	<0.001	1.47 (1.40, 1.55)	<0.001
10–12	3.05 (2.94, 3.17)	<0.001	3.01 (2.90, 3.13)	<0.001	2.96 (2.85, 3.07)	<0.001
Sex, *n* (%)						
Male	1.00 (ref)		1.00 (ref)		1.00 (ref)	
Female	1.07 (1.04, 1.10)	<0.001	1.08 (1.05, 1.11)	<0.001	1.10 (1.07, 1.13)	<0.001
Location, *n* (%)						
Urban	1.00 (ref)		1.00 (ref)		1.00 (ref)	
Rural	1.43 (1.38, 1.47)	<0.001	1.37 (1.33, 1.41)	<0.001	1.35 (1.31, 1.39)	<0.001
Country-level city	0.96 (0.92, 1.00)	0.06	0.97 (0.93, 1.01)	0.102	0.96 (0.92, 1.01)	0.089
Cognitive status, *n* (%)						
All correct	1.00 (ref)		1.00 (ref)		1.00 (ref)	
Not all correct	1.37 (1.32, 1.41)	<0.001	1.34 (1.29, 1.38)	<0.001	1.31 (1.27, 1.36)	<0.001
All wrong	6.96 (6.13, 7.91)	<0.001	6.10 (5.36, 6.95)	<0.001	4.03 (3.50, 4.65)	<0.001
Significant increase in hand washing frequency, *n* (%)						
Yes	1.00 (ref)		1.00 (ref)		1.00 (ref)	
No	7.12 (6.67, 7.59)	<0.001	6.76 (6.33, 7.22)	<0.001	4.95 (4.62, 5.32)	<0.001
Mask wearing status, *n* (%)						
Always	1.00 (ref)		1.00 (ref)		1.00 (ref)	
When in crowed places or on public transportation	1.09 (1.05, 1.13)	<0.001	1.16 (1.12, 1.21)	<0.001	1.09 (1.05, 1.13)	<0.001
Occasionally	6.02 (5.49, 6.60)	<0.001	5.75 (5.24, 6.32)	<0.001	3.65 (3.30, 4.04)	<0.001
Never	18.38 (14.65, 23.05)	<0.001	16.96 (13.43, 21.42)	<0.001	8.16 (6.29, 10.59)	<0.001

Abbreviations: CI, confidence interval; OR, odds ratio; Model 1 was adjusted by age, sex, location; Model 2 was further adjusted by cognitive status, increase hand washing frequency, mask wearing status was based on model 1.

## Data Availability

The data supporting the findings of this study are available from the corresponding author, Xiaoli Fu, upon reasonable request.
